# Differential expression of aristaless-like homeobox 4: a potential marker for gastric adenocarcinoma 

**Published:** 2016

**Authors:** Fariba Ghasemvand, Navid Nezafat, Saeed Hesami Tackallou, Daruosh Momenzadeh, Saeid Rahmanzadeh

**Affiliations:** 1 *Enzyme Technology Lab, Genetics & Metabolism Research Group, Pasteur Institute of Iran, Tehran, Iran*; 2 *Department of Pharmaceutical Biotechnology, Faculty of Pharmacy and Pharmaceutical Sciences Research Center, Shiraz University of Medical Sciences, Shiraz, Iran*; 3 *Department of Biology, Garmsar Branch, Islamic Azad University, Garmsar, Iran.*; 4 *Brain and Spinal Injury Research Center, Tehran University of Medical Sciences, Tehran, Iran *

**Keywords:** Cancer stem cell, MKN-45, ALX-4, qRT-PCR

## Abstract

**Aim:**

The objective of this experiment was to evaluate the ALX-4 mRNA expression level in different stages of human gastric adenocarcinoma compared to the gastric cancer stem cells (GCSC) and gastric cancer cell line, MKN-45.

**Background:**

Gastric cancer is the second most common cancer in the world today, leading approximately to 3-10% of all cancer-related deaths. Identification of specific biomarkers could be a crucial approach to improve diagnosis and treatment of this cancer type. Recent findings emphasized on the up-regulation of *Aristaless-Like Homeobox 4* (*ALX-4*) gene expression in several tumors.

**Material and Methods:**

MKN-45 cell culture was prepared, and gastric cancer stem cell (GCSC) isolation was performed by flowcytometry. Then, 37 fresh gastric tissue samples from cancer patient were subjected for expression analysis by quantitative RT-PCR, prior to any therapeutic intervention in the comparative study for evaluation of *ALX-4* gene expression.

**Results:**

GCSCs with cuboidal shape as well as a positive expression of CD105, CD44, CD90 and negative for CD45, CD34 markers were identified. Overexpression of *ALX-4 *was detected in 46% (3.351±2.94, P<0.05) of gastric cancer tissue specimens and GCSCs (4.31±0.04, P<0.005). The mRNA expression level of *ALX-4* in MKN-45 gastric cancer cell line was 2.81±0.07 (P<0.005). We determined that *ALX-4* mRNA level significantly correlated with the tumor grade (P=0.004), stage (p=0.000153), but not gender (P= 0.06).

**Conclusion:**

These results documented the important role of *ALX-4* in GCSCs, as an oncogene in progressive cancer, and valuable target in the treatment of drug resistant tumors.

## Introduction

 Gastric malignancy is the second most leading cause of cancer death worldwide; thus it is reported that 3-10% of all cancer-related death is due to this type of malignancy (1-3). Comparative studies among the Asian and Western countries indicated significant variations in the incidence and survival rate of gastric cancer, suggesting ethnic-related factors as a potential risk indicator (2-6). Thus, the American National Institute of Health (NIH) has classified the gastric cancer patients in three distinctive ethnic groups; those with high age-adjusted incidence (including Japanese, Koreans, Vietnamese, Native American, and Hawaiian), intermediate (Chinese, Latino and Black), and low age-adjusted incidence (Filipino and White) (7,8). Curiously, gastric cancer is one of the most prevalent types of malignancy In Iran (9). Despite many advances in surgery and adjuvant therapies, the rate of gastric cancer mortality is still significant. It is estimated that 90% of all gastric tumors are malignant, out of which 95% of cases are classified into gastric adenocarcinoma subtype (4). Almost 80-90% of gastric adenocarcinoma patients are diagnosed while the tumor has locally been advanced or spread to the other organs (7). Lack of early detection can lead to malignancy progression and consequently death. Thus, more than 80% of gastric cancer patients are generally detected in advanced stages (9, 10).Similar to the others, gastric cancer generally is complex of heterogenic cells with different histopathology and molecular fates, causing hard interpretation and diagnosis of this cancer (8). Hence, several investigations are currently undergoing to understand the molecular genetics and epigenetic aberrations contributing to the gastric cancer, and subsequently find an early diagnostic biomarker for this disease.

Aristaless-like homeobox 4 (ALX4), as a crucial member of ALX proteins family in vertebrates, plays remarkable role in neural tube closure, limb, and most particularly features of craniofacial developments (11). Dysfunction of this protein could lead to several abnormalities, including parietal foramina and front nasal. Although, investigations highlighted the essential role of* ALX* genes family in the developmental process and their potential interactions with each other in human and rodents, the precise mechanism of action remains to be elucidated (11). It has been reported that ALX4 is expressed in the mesenchymal cells of limbs, bones, teeth, hair, whiskers and mammary gland during the development (12–14). Targeted mutation of *ALX4* resulted in mice with multiple abnormalities, such as poly dactyly, defects on craniofacial structure, and body wall closure (12, 15). Further investigations showed that loss of ALX4 function in human caused defects in the craniofacial development (16, 17). In addition to the critical function of ALX4 in development, recent studies have reported the relation of ALX4 expression with cancer. Thus, hypermethylation of the *ALX4* gene was correlated with tumorigenesis and prognosis in colorectal cancer (18). In lung cancer, the ALX4 expression was silenced by hypermethylation, and ectopic expression of this protein could inhibit proliferation of lung cancer cells in vitro and in vivo (19). In contrast, ALX4 was remarkably expressed in a subtype of medulloblastoma, as the most common pediatric brain tumor (20). Investigations have introduced HOXB13/SLUG and ALX4/SLUG axes, as two novel pathways, promoting EMT and invasion of lung and ovarian cancer cells (21, 22).

 In this study, we evaluated *ALX-4* mRNA expression levels in 37 samples of gastric cancer tissue with different development grads, in comparison with respective normal gastric tissue, derived from the tumor peripheral normal regions. In addition, *ALX-4* gene expression was investigated in gastric malignant cell lines (MKN-5), as well as gastric cancer stem cells (GCSCs). 

## Material and Methods

**Study population**

The study was approved by the ethics committee of Shiraz University of Medical Sciences and the written informed consent for the participation in the study was obtained from each subject at the cancer department in Imam Khomeini and Labbafinejad Hospitals. All gastric cancer tissue specimens were obtained from 37 patients, aged 29–57 years old (mean, 38 ± 9 (±SEM)) who had not received any treatment prior to the surgery (Table 1). Malignancy was subsequently confirmed by postoperative pathological studies. Upon tissue biopsies, they were transported in RNAlater RNA Stabilization Reagent (Qiagen, USA) at 4°C. They were then washed with PBS, sliced into small pieces, and stored at −80°C until use.

**Table 1:** Histological and clinical data of the biopsied patient tissues

Low *ALX-4* (n=4)	Medium*ALX-4*(n=16)	High *ALX-4* (n=17)	Total (n=37)	
2/2	12/4	10/7	21/16	Men/Women
--- --- 4	5 7 4	12 3 2	17 10 10	Tumor grade I II III
--- 2	4 12	16 3	20 17	Tumor stage I/II III/IV

**MKN-45 Cell culture**

The human gastric adenocarcinoma cell line, MKN-45 (NCBI NO: C615), was obtained from Pasteur Institute of Iran, and cultured in RPMI-1640 medium supplemented with 10% fetal bovine serum (FBS) (Sigma Aldrich, St. Louis, MO), 100 µg/ml streptomycin and penicillin (Sigma Aldrich, St. Louis, MO) in a humidified incubator (Binder, Germany), containing 5% CO_2 _at 37˚C.

**Cell sorting For CD44 Posetive cell**

In this study, MKN-45 cells were sorted for CD44 surface marker as previously described by Gao, et al. (23). Briefly, CD44 monoclonal antibody was added to MKN-45 cells and they were passed through a MACS column (Miltenyi Biotec, Auburn, Calif., USA). Thus, the human gastric adenocarcinoma cell containing CD44^+^surface markers was separated using the other cells. These cells (known as GCSCs) were then cultured as previously described and prepared for further investigations.

**Flow-cytometry analysis for stem cell surface marker**

To investigate GCSCs, these cells were isolated from MKN-45 (10^5^-10^6^ cells; passage 2) using CD44^+^ surface marker flow-cytometry. In this experiment, the cells were initially washed by HBSS+2%BSA two times, followed by incubating with the specific primary antibodies (diluted with PBS) at recommended manufacturer’s concentrations. Different antibodies were used at this stage to determine following cell surface markers: CD90, CD44, CD133, CD34 and CD45 (abcam, UK). The samples were incubated for 30 minutes and washed twice with PBS. Later, a secondary IgG antibody with fluorescent conjugates was diluted according to the manufacturer’s instruction. The cells were then incubated for 20 minutes at 4°C and analyzed with flowcytometer (Becton Dickinson, Germany).

**Immunofluorescence**

About 2x10^4^ cells were seeded on a 4-well Lab-TekII Chamber Slide. After 24 hours, the cells were washed with PBS twice and fixed in 2% paraformaldehyde. The cells were subsequently permeabilized with 0.1% Triton X100 diluted in PBS buffer, by incubation at 40˚C for 30 minutes. This procedure was followed by three times washing in PBS and cell induction with the blocking solution (10% goat serum in PBS). The cells were then incubated with the primary antibodies (2 hours to overnight), and washed 3 times with PBS plus 0.1% Tween-20 for 15 minutes. They were incubated with secondary antibodies (abcam, UK) for 2 hours. The slides were ultimately washed extensively with PBS and mounted with slow fade Light Anti fade Kit (Invitrogen, UK). All samples (including controls and tests) were photographed using an immunofluorescence microscope (LabPro CETI, OXFORD, UK) and identical exposure times. 

**Quantitative RT-PCR**

RNA was extracted from the indicated cell lines and tissues, using a Qiagen RNeasy kit (Qiagen, USA). Complimentary DNA (cDNA) was synthesized from 1µg of RNA, according to the instructor’s guideline (Takara, Japan). The quantitative RT-PCR(qRT-PCR) was performed in a final volume of 20 µl, using 2µl cDNA, 1x SYBR Green PCR Master Mix (Takara, Japan) and appropriate primer sets, on a Rotor-Gene Qreal-time thermocycler (Qiagene, USA). Specific primers for each *ALX-4*and *B-ACTIN *mRNA genes were designed by AlleleID software 6.0 (PREMIER Biosoft, CA, USA), as shown in the table 2. Thermal cycling was composed of incubation at 95°C for 10 minutes, followed by 40 cycles of 95°C for 15 seconds, 57°C for 30 seconds, and 72°C for 45 seconds. Data were subsequently normalized to *ALX-4 *expression applying the comparative threshold cycle method. The PCR efficiencies for *ALX-4* and *B-ACTIN *were verified by generating relevant standard curves. The relative level for *ALX-4* gene expression was compared based on fluorescence intensity changes of samples from cancer versus compliant normal tissues. All experiments were performed in triplicate. More than two-fold increase in expression was considered as up-regulation, while a more than two-fold decrease was considered as down-regulation. The range between these two values was interpreted as no change or normal expression. 

**Immunohistochemistry:**

Immunohistochemistry (IHC) was performed as previously described (24). Paraffin embedded tissue sections were deparaffinized and rehydrated. Slides were treated with 3% H_2_O_2_ for 10 minutes and 2% BSA for 30 minutes at room temperature, followed by adding 2 µg/ml monoclonal Anti-ALX-4 antibody (abcam, UK) and overnight incubation at 4 °C . Later, the slides were washed three times with PBS and stained with HRP-linked anti-mouse secondary antibody (abcam, UK) for 30 minutes at room temperature, and then washed 3 times with PBS, followed by chromagen detection with DAB for 10 minutes, and hematoxylin and eosin counterstaining.

**Table 2:** QRT-PCR primer sequence

Tm (°C)	Product length	Sequence (5'->3')	
60	264bp	Forward primer: GCAAAGCTAGAATTCGGCGG Reverse primer: GAATACTCGCCGTAGGGGTG	*ALX-4*
59	115bp	Forward primer: CCCTTCATTGACCTCAACTACATG Reverse primer: GGGATTTCCATTGATGACAAGC	*B-ACTIN*

**Western-blot Analysis**

The samples were collected and lysed with standard RIPA buffer (24, 25). After centrifugation at 40000g, 4°C for 45 minutes, the supernatant was applied for SDS-PAGE analysis.

Then, 100μg of protein from each tissue sample (n =2), CD44+ GCSCs (n=1) and MKN-45 gastric cancer cell line (n=1), was subjected to 12% SDS–PAGE. Proteins were transferred to the nitrocellulose membrane (Millipore, USA) using Western-blot technique, followed by blocking with 5% bovine serum albumin (BSA; Sigma, Germany) at room temperature with shaking for 60 minutes. The membranes were then incubated with mouse anti-ALX-4 (abcam, UK) antibody (1:1000 dilutions) for 1 hour followed by the incubation of membranes with HRP conjugated anti-mouse antibody at room temperature with mild shaking. Enhanced chemiluminescence (ECL) western-blotting system (GE Healthcare, USA) was used to develop the membrane on high a performance chemiluminescence film (GE Healthcare, USA) according to the manufacturer’s guidelines. After each step, the membrane was washed with PBS.

**Statistical analysis**

Data was analyzed using the Microsoft office Excel. The correlation between *ALX-4* mRNA expression level of various samples were analyzed by Pearson’s correlation. The association between gene expressions was also evaluated by student t-test. P value of less than 0.05 was considered statistically significant. 

## Results

**Figure 1.**
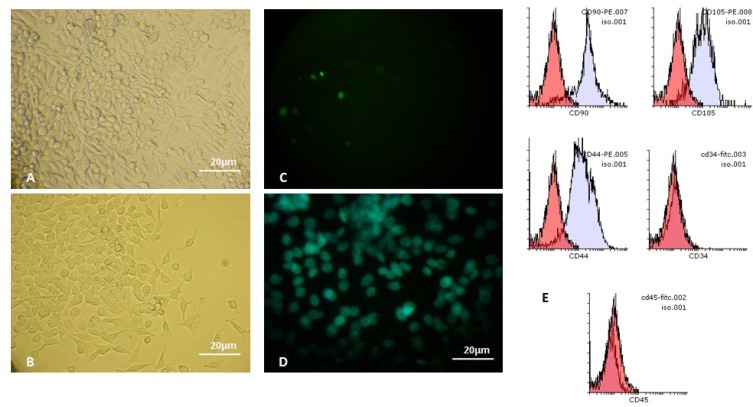
Comparing MKN-45 and relevant CD44^+^ GCSCs. Images show morphology of (A) MKN-45 cell line, (B) CD44^+^GCSCs, and ICC staining for ALX4 protein in (C) MKN-45 cell line,(D) GCSCs. Image (E) show positive expression of CD44, CD90, CD105 and negative expression of CD34 and CD45 surface markers in theCD44^+^GCSCs. Red color graph indicates the negative area of diagram and blue color shows positive area. Scale bar: 20µm

In this study, we initially sorted MKN-45 gastric adenocarcinoma cells, in terms of determining the cells with CD44 surface marker. Thus, the CD44^+^ MKN-45 cell line was considered as GCSCs, due to observing several properties of stem cells. Morphologically, CD44^+ ^GCSCs showed different appearances compared to the MKN-45 cell line (Fig. 1-A and -B). CD44^+ ^GCSCs were generally evaluated as cuboidal-shaped cells with scant cytoplasm and granules around the nuclei (Fig. 1B). Curiously, immunocytochemistry (ICC) staining showed high expression of the ALX-4 protein in GCSCs, while this protein was presented remarkably less in MKN-45 cell line (Figure 1-C and-D). Data analyses obtained from flow-cytometer revealed that GCSCs highly expressed CD44, CD105 and CD90 surface markers, while they did not express CD45 and CD34 (Fig. 1-E). This study confirmed findings obtained in 2013 (26), implicating purification of stem cells from gastric cancer.

***ALX-4*** **gene expression in gastric cancer cells and tissues**

**Figure 2:**
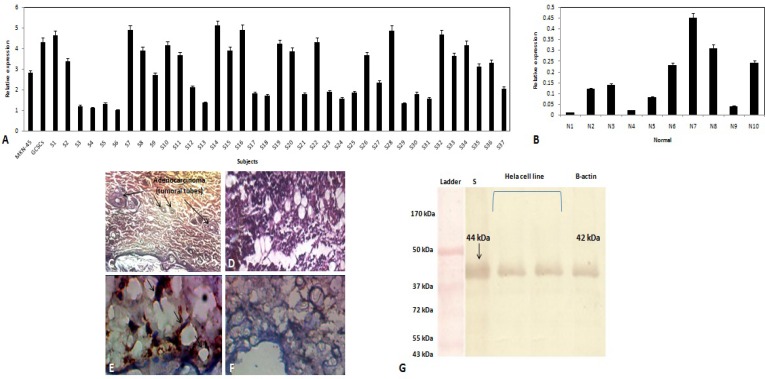
ALX-4 expression analysis.Relative *ALX-4*mRNA expression level of (A) MKN-45 cell line, CD44^+^GCSCs and malignant gastric tissues (n=37), and (B) normal gastric tissue samples (n=10).Hematoxilin and eosin staining of (C) gastric cancer and (D) normal tissues.Immunohistochemistry staining of ALX-4 protein in (E) gastric cancer and (F) normal tissues.(G) Expression of ALX-4 was also detected by Western-blotting in gastric cancer tissues. S: cancer sample; HeLa cell line was used as positive control. B-CTIN was applied as housekeeping protein. The results are presented as mean±SEM of mRNA expression P < 0.05. Magnification 60x

The mRNA expression level of *ALX-4*gene was analyzed in MKN-45 cell line, CD44^+^GCSCs, as well as malignant and normal gastric tissue samples, using qRT-PCR. Relative data analysis showed a higher expression of the *ALX-4*mRNA in GCSCs compared to MKN-45 cells (Fig. 2-A). In addition, we determined that the *ALX-4*mRNA expression was up regulated in malignant in contrast to normal gastric tissues (Fig. 2-A and -B). Hematoxilin and eosin staining of gastric cancer (Figure 2C) and normal tissues (Figure 2D). Immunohistochemistry (IHC) analysis showed accumulation of ALX-4 protein in malignant gastric tissue (Fig. 2-E) while this protein was not observed in normal gastric tissue (Fig. 2-F). These findings were also confirmed using Western-blot analysis. Thus, malignant gastric tissues showed *ALX-4*mRNA expression, while no expression was observed in normal gastric tissues (Fig. 2-G). 

**Correlation of** ***ALX-4*** **gene expression and malignancy stage**

In this study, we evaluated the mRNA expression level of the *ALX-4* in gastric cancer tissues, gastric cancer stem cell and MKN-45 cell line. In addition, the clinical significance of this mRNA gene, as a potential prognostic factor, was evaluated. Findings showed that 45.94% of gastric cancer patients highly expressed *ALX-4*(>3.5 fold), 43.24% expressed moderately (1.5-3.5 fold), and 10.81% of the patients showed low expression of this mRNA gene (<1.5 fold), compared to the control group. These findings suggest that ALX-4 is up-regulated in most of the gastric cancer patients. Therefore, we further investigated the relationship of this up-regulation with each of tumor stage, grade and gender in gastric cancer patients (Fig. 3). In this study, 54% of the cases (20 patients) were diagnosed with stages I/II malignancy progression, while 46% (10 patients) were in stages III/IV. Findings demonstrated higher *ALX-4* mRNA expression level in the stages I/II malignant tissues (mean expression level=3.934, STDEV= 0.855, α=0.05, p= 0.000153), in contrast to stages III/IV (mean expression level=1.762, STDEV=0.63, α=0.05, p= 0.001).

Moreover, analyses of 17, 10 and 10 patients with grades I, II and III, respectively demonstrated that *ALX-4* mRNA expression level is down-regulated by raising the grade of the disorder. Thus, the mean expression level of *ALX-4* was 4.17 for grade I (STDEV= 0.596, α=0.05, p= 0.004), 2.25 for grade II (STDEV= 0.88, α=0.05, p= 0.004) and 1.61 for grade III (STDEV= 0.431, α=0.05, p= 0.004). In contrast, comparing 21 male and 16 female patients revealed no significant gender-based difference. In addition, no significant correlation was observed between the *ALX-4* mRNA expression level and any of tumor stage, grade or gender in the control group. 

## Discussion

Gastric carcinoma is one of the most prevalent malignant cancers in the world, often associated with poor prognosis (11, 13). Metastasis and tumor recurrence are two major obstacles to the long-term survival of gastric carcinoma patients (4, 9, 10).

Early interposition with treatment is very momentous to catch survival profit (10, 16). Therefore, better understanding of molecular biology events in gastric cancer metastasis and recurrence is required (2, 4).

Here, we reported that high level of *ALX-4* mRNA and protein expressions could potentially be a novel biological marker correlating with early stages of the gastric cancer development.

ALX4 protein, as a member of the homeobox protein family, directs formation of body structures during early embryonic development (13, 14). ALX4 protein is necessary for normal development of the head and face, particularly the formation of the nose, starting around the fourth week of embryo development (15, 16). This protein is also involved in the formation of different skin layers (16, 18), however the role of this process is poorly understood. ALX4 protein is a transcription factor, which regulates the activity of several genes involved in cell growth, division (proliferation), and movement (migration), controlling cell growth in particular times (18, 19).

The above observations suggested CD44^high^ stem-like cells could be generated from more differentiated populations of normal mammary epithelial cells by inducing an EMT. By extension, we speculated that EMT could promote the generation of cancer stem cells from more differentiated neoplastic cells.

In addition, we determined that *ALX-4* mRNA expression level was associated with the clinical characteristics (such as grade, stage) in gastric cancer tissues. These findings are in agreement with previous report, indicating that ALX-4 can play oncogenic role in different malignancies, like medulloblastoma (20). Ushiku, et al. proposed that oncogenic protein up-regulations were correlated with older age, male sex, intestinal-type histology, and synchronous hepatic metastasis in gastric carcinoma (27). Accordingly, our findings were compatible to this report implicating non-association of *ALX-4* mRNA level with gender in patients group. Although, these data proposed that *ALX-4* expression in gastric cancer tissues has a higher degree of tumor complexity. Previous data generally focused on the role of ALX-4 on carcinogenesis and treatment of malignant cell lines and tissues (20,28,29). It has been demonstrated that cell culture studies could not fully address the role of the microenvironment on internal and external molecular cascades in malignant cells, and it could be different even in various cell lines (13,14, 25). The role of*ALX-4* expression was evaluated in CD44^+^GCSCs, isolated from MKN-45 gastric adenocarcinoma cell line, to determine the therapeutic effect of this protein, particularly for drug resistant tumors (25, 27).

In the present study, our findings showed that *ALX-4* mRNA level in gastric cancer patients were significantly higher compared to the control group. In addition, we investigated the interrelationship of the *ALX-4 *mRNA expression level and malignancy stage, grade, tumor recurrence and metastasis, as well as overall survival rate in Iranian gastric cancer patients. Curiously, the mRNA expression level of this gene was determined to be higher in early stages of malignancy, suggesting the crucial role of ALX-4 protein in prognosis of gastric cancer. Similarly, these results showed that the *ALX-4 *mRNA expression level was reduced with developing tumor grades. While no significant difference was observed on the *ALX-4 *mRNA expression level, between male and female patients. Analyses of data suggested no correlation of ALX-4 with tumor stage, grade or gender in controls, when a significant difference was observed by comparing malignant tissues and control groups. To the best of our knowledge, the role of this protein in tumor progression is still controversial (27-29), while it has not been well elucidated yet in gastric cancer cells and GCSCs. In this study, we determined that ALX-4 is significantly up-regulated in most of the gastric cancer cases.

In this experiment, we determined that *ALX* gene family could activate particular pathways regulating gastric cancer cell phenotype. These EMT regulators may play an important role in cancer progression. The mRNA expression of *ALX-4* associated with gastric cancer invasion and resistance to target therapy. Regarding over-expression of *ALX-4 *in gastric cancer stem cells and malignant gastric tissues, this gene may play a critical role in the maintenance of the tumor phenotype. This is compatible to the previous reports, which implicated the preservative role of ALX-4 in tumor phenotype (4, 16, 25), suggesting an oncogenic effect for this protein. However, our results suggested that there might be some relationship between ALX-4 expression and early grade or stage of gastric cancer development, raising the potential prognostic capacity of this protein to diagnose early stage/grade gastric tumors. Ultimately, the oncogenic role of ALX-4 shows while it may be an important prognostic factor, this protein may be used as a viable therapeutic target for inhibiting malignant cell progression. More studies will be necessary accompanied by with very specimens for confirmation role of ALX-4 gene in oncogenic, EMT and early diagnoses marker.
